# Liquid biopsy in lung cancer: significance in diagnostics, prediction, and treatment monitoring

**DOI:** 10.1186/s12943-022-01505-z

**Published:** 2022-01-20

**Authors:** Wen Li, Ji-Bin Liu, Li-Kun Hou, Fei Yu, Jie Zhang, Wei Wu, Xiao-Mei Tang, Feng Sun, Hai-Min Lu, Jing Deng, Jie Bai, Juan Li, Chun-Yan Wu, Qin-Lu Lin, Zhong-Wei Lv, Gao-Ren Wang, Geng-Xi Jiang, Yu-Shui Ma, Da Fu

**Affiliations:** 1 National Engineering Research Center of Rice and Byproduct Deep Processing, College of Food Science and Engineering, Central South University of Forestry and Technology, Changsha, 410004 Hunan China; 2 Institute of Oncology, Affiliated Tumor Hospital of Nantong University, Nantong, 226631 Jiangsu China; 3 Department of Pathology, Shanghai Pulmonary Hospital, Tongji University School of Medicine, Shanghai, 200433 China; 4 Department of Nuclear Medicine, Shanghai Tenth People’s Hospital, Tongji University School of Medicine, Shanghai, 200072 China; 5 Department of Immunology, School of Medicine, Nantong University, Nantong, 226019 Jiangsu China; 6 General Surgery, Ruijin Hospital & Institute of Pancreatic Diseases, Shanghai Jiaotong University School of Medicine, Shanghai, 200025 China; 7 Department of Thoracic Surgery, Affiliated Tumor Hospital of Nantong University, Nantong, 226631 Jiangsu China; 8 Department of Radiotherapy, Affiliated Tumor Hospital of Nantong University, Nantong, 226631 Jiangsu China; 9 Department of Thoracic Surgery, Navy Military Medical University Affiliated Changhai Hospital, Shanghai, 200433 China

**Keywords:** Lung cancer, Diagnostics, Liquid biopsy, CTCs, ctDNA, Exosomes

## Abstract

Primary lung cancer is one of the most common malignant tumors in China. Approximately 60% of lung cancer patients have distant metastasis at the initial diagnosis, so it is necessary to find new tumor markers for early diagnosis and individualized treatment. Tumor markers contribute to the early diagnosis of lung cancer and play important roles in early detection and treatment, as well as in precision medicine, efficacy monitoring, and prognosis prediction. The pathological diagnosis of lung cancer in small biopsy specimens determines whether there are tumor cells in the biopsy and tumor type. Because biopsy is traumatic and the compliance of patients with multiple biopsies is poor, liquid biopsy has become a hot research direction. Liquid biopsies are advantageous because they are nontraumatic, easy to obtain, reflect the overall state of the tumor, and allow for real-time monitoring. At present, liquid biopsies mainly include circulating tumor cells, circulating tumor DNA, exosomes, microRNA, circulating RNA, tumor platelets, and tumor endothelial cells. This review introduces the research progress and clinical application prospect of liquid biopsy technology for lung cancer.

## Introduction

Primary lung cancer is a common malignant tumor in China. It is divided primarily into two types: small cell lung cancer (SCLC) and non-small cell lung cancer (NSCLC). NSCLC accounts for about 85% of all lung cancer types [1] and is a highly heterogeneous population that is divided into lung adenocarcinoma (LUAD) and lung squamous cell carcinoma (LUSC). Most of the patients have signs of metastasis when symptoms appear, which is the main reason for the high mortality rate. Therefore, early diagnosis and early treatment would be an effective measure to reduce the mortality rate among patients with primary lung cancer.

Tumor markers contribute to the early diagnosis of lung cancer and play an important role in early detection and treatment, as well as precision medicine, individualized treatment, and prognosis prediction. Low-dose spiral CT (LDCT) is considered a standard method for the early diagnosis of lung cancer, which can significantly improve the survival rate of lung cancer patients [2]. However, LDCT has some disadvantages, including high false-positive rate and radiation exposure.

**Fig. 1:**
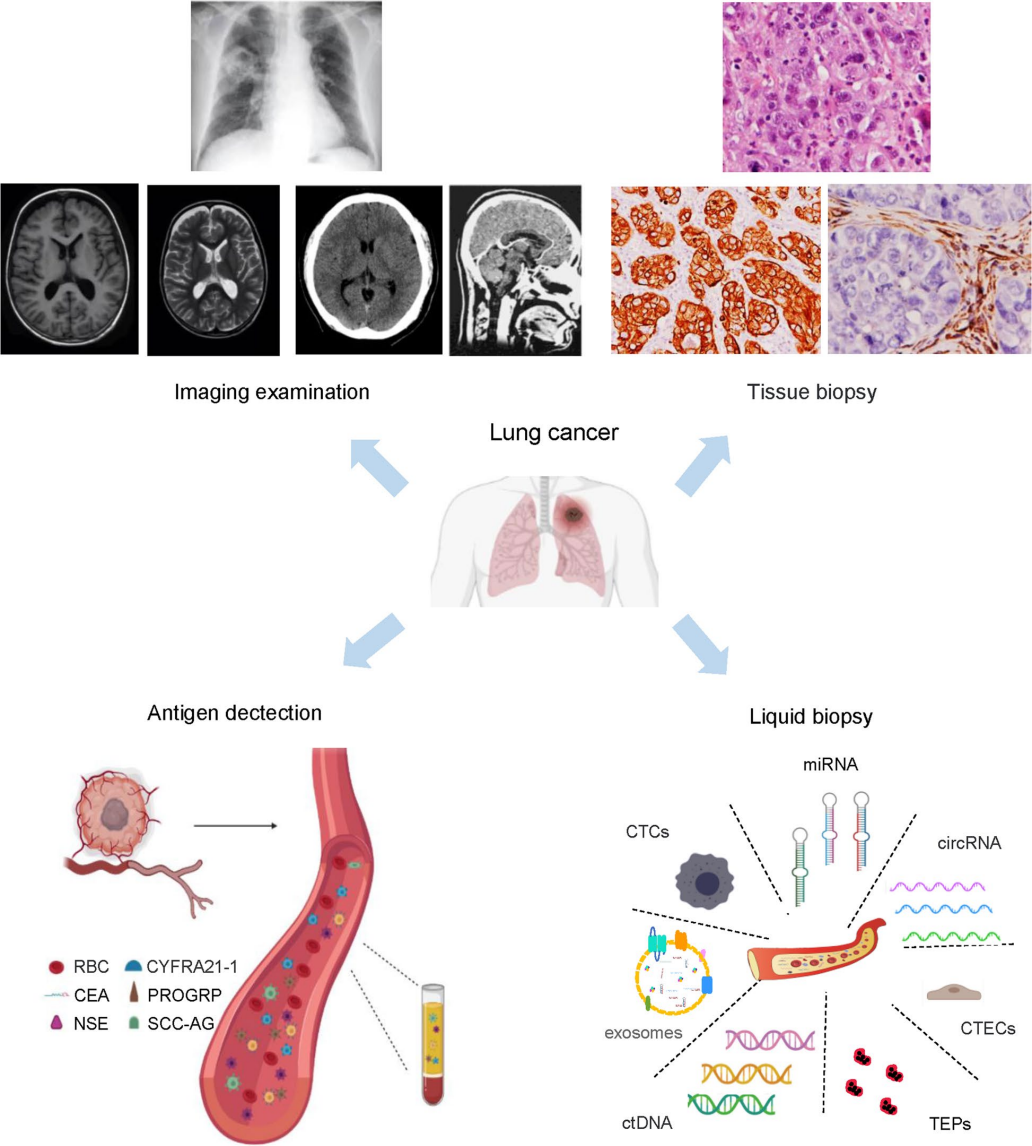
Clinical examination and diagnosis for lung cancer. Routine imaging examination for early examination and diagnosis of lung cancer includes X-ray, magnetic resonance imaging, and low-dose spiral computed tomography (CT). Vital approaches used to examine cancer tissue specimens include H&E staining, immunohistochemistry, and optical imaging modalities. Examination and diagnosis of peripheral blood with tumor antigens is routinely used in clinical examination. Liquid biopsy is emerging as a promising method for the identification of patients with a high risk of disease progression after curative surgery, as well as longitudinal monitoring for disease progression and therapy response

Besides, An clinical diagnosis requires a solid biopsy in order to determine tumor histology and staging. However, the operation to collect tissue biopsies has certain limitations and risks. The role of conventional biomarkers is largely limited by spatial and temporal tumor heterogeneity whereas liquid biopsy seems to be promising to circumvent tumor heterogeneity to identify candidate patients who may response to therapy, to dynamically monitor treatment effect and to unveil resistance mechanism. Liquid biopsy is non-invasive way to easily obtain tissue specimens from patients. This biopsy method allows for real-time monitoring of the tumor, which make it the most promising substitute for tumor tissue specimens in the clinic (Fig. 1).

In a broad sense, liquid biopsy is the process by which blood or bodily secretions are tested for cancer cells. Blood is the most commonly tested liquid biopsy in the clinic. Liquid biopsies help further clarify the characteristics of lung cancer by identifying tumor cells or tumor DNA released into the blood by the growth and/or apoptosis of cancer cells. Therefore, blood detection, a highly sensitive technology, has the ability not only to diagnose cancer but also to reveal key characteristics of the tumor, such as the type of cancer and whether the cancer cells have major gene mutations.

During the past 5 years, the management of lung cancer has been drastically improved with the application of immunotherapy, especially after the advent of immune checkpoint inhibitors (ICIs). Despite that ICIs have resulted in significant therapeutic benefit and prolongation of survival in the whole, the extent of benefit in ICI is not uniform. Not all patients benefit from immunotherapy and most patients would eventually experience disease progression. Although immunotherapies are largely thought to have fewer adverse effects than chemotherapy and molecular targeted therapy, the immune-related adverse events such as myocarditis and thyroiditis could be lethal and it’s thus crucial to identify patients who are at risk of therapy related toxicity. Moreover, hyper progressive disease (HPD) after treatment with ICIs could make the condition much worse. Thus, predictive biomarkers of ICI response are urgently needed to guide treatment decision and patient selection, as well as to better understand and overcome mechanisms of resistance. With the application of liquid biopsy, it’s able to reveal a much more comprehensive tumor genome and dynamically monitor the genetic change along treatment. In general, liquid biopsy could help select candidate patients who would benefit from immunotherapy in several aspects.

**Fig. 2:**
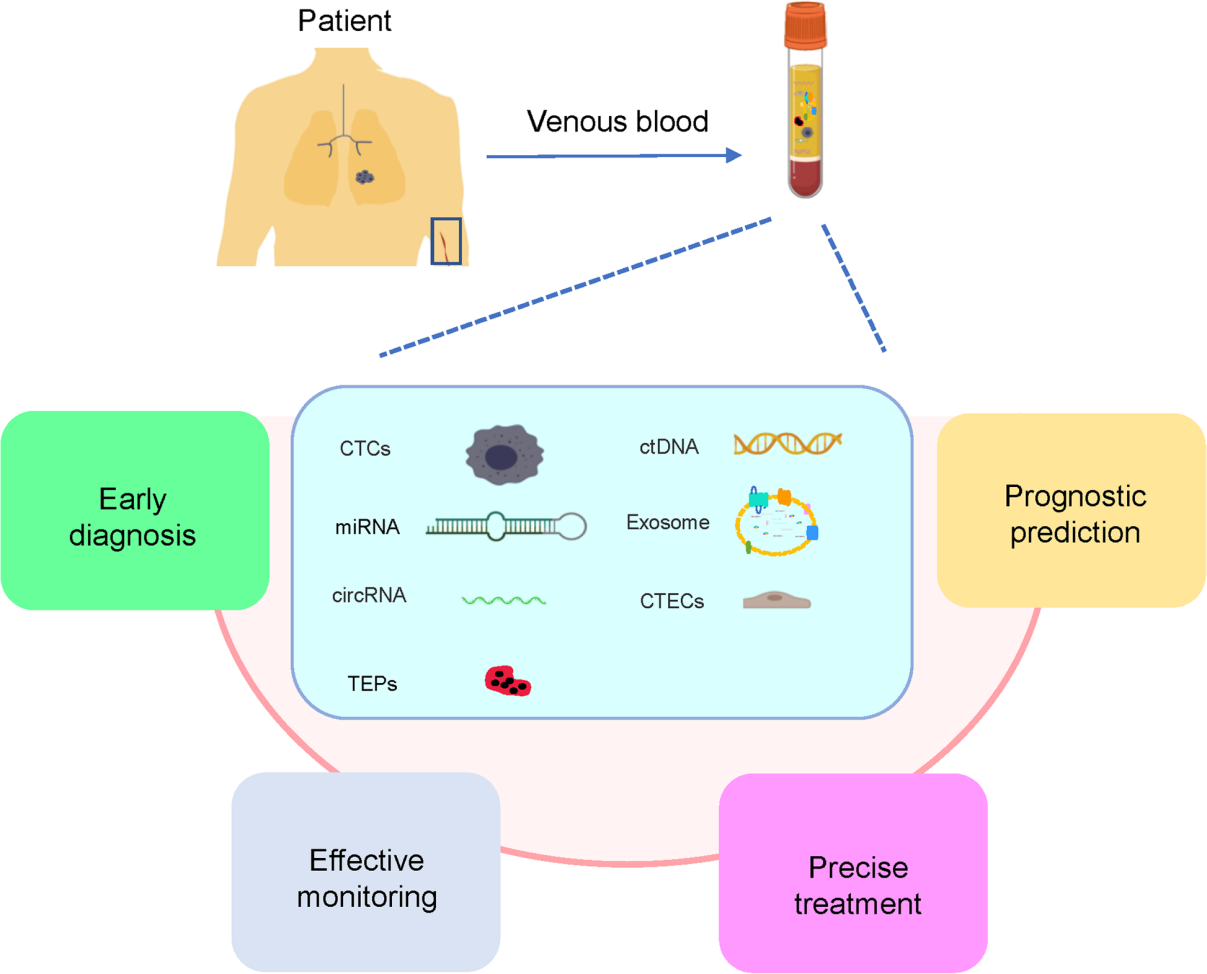
Clinical application of liquid biopsy in lung cancer. Circulating tumor cells (CTCs), circulating tumor DNA (ctDNA), exosomes, microRNAs (miRNAs), circular RNAs (circRNAs), tumor-educated platelets (TEPs), and circulating tumor vascular endothelial cells (CTECs) in the venous blood from lung cancer patients(e.g., liquid biopsy) have the potential to be used clinically to provide unique opportunities for the real-time monitoring of disease progression and treatment response, in addition to studying tumor heterogeneity

At present, liquid biopsy typically detect circulating tumor cells (CTCs), circulating tumor DNA (ctDNA), exosomes, microRNAs (miRNA), peripheral blood circulating RNA, tumor-educated blood platelets (TEPs), and circulating tumor vascular endothelial cells, CTECs [3], among which CTCs, ctDNA, and exosomes are the most commonly detected biomarkers (Fig. 2). In this review, the clinical application of liquid biopsy technology in lung cancer, including early diagnosis, individualized treatment, prognosis prediction, and the research progress of related technology, are summarized.

### Clinical application and technical research progress of liquid biopsy in lung cancer

#### CTCs

The concept of CTCs was first proposed by an Australian doctor in 1869 [4]. CTCs refer to tumor cells that fall off from primary or metastatic lesions and enter the peripheral blood either spontaneously or as a result of a diagnosis or treatment operation. Apoptosis or phagocytosis of the tumor cells then occurs in the peripheral blood. However, a few tumor cells may escape apoptosis/phagocytosis and undergo epithelial mesenchymal transformation (EMT), thereby acquiring stronger fluidity, invasiveness, and increased ability to adhere and penetrate the vascular wall, resulting in distant metastasis.

CTCs can be detected in the blood of patients with distant metastasis, and CTC analysis offers several benefits, including analysis of the tumor morphological or genetic information, genetic analysis of the tumor genotype, improvements in the classification and stratification of patients, effective monitoring of the patient’s response, improvements in the clinical prognosis, and achievement of the goal of personalized treatment by providing specific drugs suitable for each patient’s tumor [5]. A large number of studies have found that CTCs can provide early diagnosis, predict the risk of metastasis, recurrence, and progression, and monitor and identify drug treatment in realtime. An efficient CTC separation method not only detects small tumors in the early stage, but is also used for the diagnosis of early and advanced cancer patients. Additionally, evaluation of mutations in the CTCs will help regulate and monitor patient treatment in realtime, which provides a strong prospect for the treatment of lung cancer in the future.

**Fig. 3:**
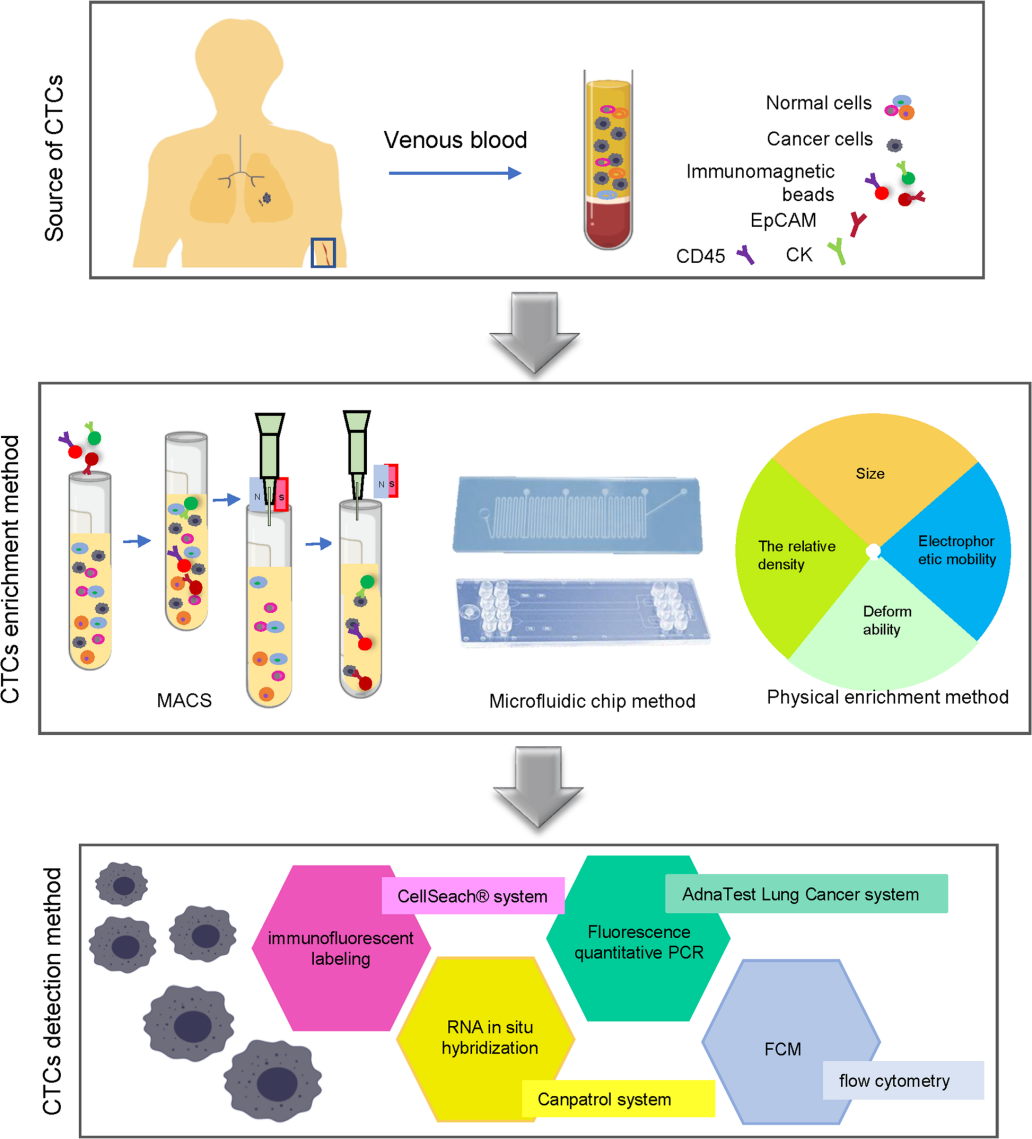
Enrichment and typical detection methods for CTCs. Working with CTCs includes the following three analytical steps: enrichment, detection, and analysis. Enrichment includes label-dependent approaches based on antibodies used for positive or negative enrichment of CTCs, as well as label-independent technologies. In the last decade, individual CTCs or CTC clusters have been analyzed downstream at the DNA, RNA, or protein level

At present, the primary methods to enrich CTCs include an immune enrichment method and a physical enrichment method. Immuno-enrichment methods can be divided into an immunomagnetic bead method and immunoadsorption method using a microfluidic chip. Immunomagnetic bead methods include both a positive-enrichment method and negative-enrichment method. CellSearch system is a representative product for the positive enrichment of CTCs based on the immunomagnetic bead method [6]. This system can automatically photograph, count, and analyze stained cells. A negative-enrichment method using immunomagnetic beads enriches CTCs by removing background blood cells. Immunoadsorption methods are based on microfluidic chips, and the processing speed of microfluidic chips commonly use the CTC chip, which is the fastest of the four chips. The physical enrichment method enriches CTCs by using differences between CTCs and blood cells, including size, relative density, and electrical characteristics. Processing the peripheral blood via the above enrichment method reduces the background blood cell levels, allowing the remaining cell populations to be detected and analyzed. At present, the detection methods of CTCs mainly include immunofluorescence labeling, RNA in situ hybridization, fluorescence quantitative PCR, and flow cytometry (Fig. 3).

The monitoring of CTCs in patients with early lung adenocarcinoma reflects the value of early diagnosis. When monitoring CTCs in patients with early lung adenocarcinoma, CTCs were divided into epithelial, mesenchymal, and epithelial mesenchymal CTCs according to EMT biomarkers. The results showed that CTC counts increased significantly in all patients with tumor progression. Compared with patients with disease-free survival, patients with tumor progression had higher post-operative CTC levels or ranges [7].

Next-generation sequencing (NGS) of CTCs was performed in patients with early lung cancer in order to identify lung cancer-related gene mutations. After NGS analysis of CTCs from lung cancer patients, more than 50% of patients were found to carry four common mutant genes (Notch1, IGF2, EGFR, and PTCH1) [8]. In addition, when analyzed by LC-MS, 100 differential metabolites were found, in which 10 different metabolites were identified to have potential clinical value in the diagnosis of CTC-positive early lung cancer, suggesting that NGS and metabonomics of CTCs may provide new tumor markers for the early diagnosis of lung cancer [9]. Additionally, CTCs were isolated from people that had a high risk of developing lung cancer (i.e., smokers with chronic obstructive pulmonary disease) who did not find nodules on a chest CT scan. A CT scan was also performed yearly in this population, and it was found that the pulmonary nodules of patients with CTCs were associated with lung adenocarcinoma. In this population, the predictive value of secondary lung cancer associated with the initial presence of CTCs was nearly 100% [10].

Some studies have shown that monitoring CTCs plays an important role in the precise treatment of lung cancer. CTC detection has been reported to be used as an alternative method in patients with anaplastic lymphoma kinase (ALK) gene rearrangement in NSCLC when appropriate tissue biopsy samples could not be obtained [11]. It has also been suggested that CTCs in a liquid biopsy compensate for the fact that a tissue biopsy cannot fully reflect the heterogeneity of tumor cells and is thus more conducive to the dynamic monitoring of disease progression. In another study, blood was collected every 2 months for CTC analysis from EGFR-mutant NSCLC patients treated with erlotinib until disease progression and found that CTCs can be used to supplement the tissue-based EGFR mutation detection in lung cancer and to guide the precision treatment of EGFR mutation when appropriate tissue biopsy samples cannot be obtained for detection [12]. Therefore, it can be inferred that the DNA detected by CTCs can predict lung cancer progression and secondary drug resistance. Thus, continuous monitoring of the therapeutic response of lung cancer via CTC analysis is of great value and research prospect.

Precision treatment is the tailoring of medical treatment to the individual characteristics of the tumor [13]. Individual differences in the response of patients to drug treatments may be caused by tumor heterogeneity, gene expression differences, and polymorphisms. CTC detection is of great value in early tumor diagnosis, as well as determining curative effects, prognosis evaluation, and individualized medical treatment. CTC analysis is convenient, non-invasive, highly repeatable, and allows for real-time monitoring and subsequent analysis and detection of DNA, RNA, and protein molecules [14, 15]. The detection of CTCs is a non-invasive biopsy, which is conducive for the screening of early diagnostic markers of lung cancer [16]. CTCs in peripheral blood are an ideal biopsy sample source that can reflect individual and tumor heterogeneity.

CTC analysis can theoretically provide more comprehensive and representative information regarding tumor deposition; however, it also has many clinical limitations. Because the content of CTCs in peripheral blood is very low, further exploration is needed in order to learn how to effectively reduce background cells, efficiently enrich CTCs, and improve the accuracy of CTC detection [17]. CTCs are generally tumor cells that fall off from the primary focus, metastasis, or surgery and enter the peripheral blood. At present, the dissemination law of CTC is not known, and the appropriate detection time cannot be determined. Different times and different treatment methods will lead to different phenotypic, genomic, and functional characteristics of CTCs, which influences its clinical application [18, 19]. In view of the above situation, CTC detection needs more effective CTC enrichment and counting methods. Using single-cell genomics and transcriptomics to analyze each CTC can more accurately obtain tumor-related information, find the dynamic changes in the tumor, analyze the law of tumor growth, provide a more accurate basis and guidance for the early diagnosis of cancer, direct research on the mechanism of recurrence, metastasis, and drug resistance, and guide individualized treatment [20]. With the development of detection technology, CTCs will be more widely used in tumor clinical diagnosis and treatment.

#### ctDNA

Cell-free DNA (cfDNA) is non-encapsulated DNA in the blood, which can be separated from plasma [21], and may be released into the blood through the apoptosis or necrosis of cells. cfDNA molecules in blood circulation are rapidly removed within 1 h of release. In the plasma of healthy people, it was found that the level of cfDNA from normal cells is low (10–15 ng/ml on average). The levels of cfDNA, however, increases under tissue stress conditions, including exercise, inflammation, surgery, or tissue damage [22]. ctDNA, small nucleic acids released by apoptotic or necrotic tumor cells, is a type of cfDNA and encodes the genes of tumor cells [23]. Most ctDNA fragments are double-stranded fragments with a length of 160–200 base pairs, which is roughly the same as the length of nucleosomes [17]. The amount of ctDNA entering the blood is affected by many factors, such as tumor location, size, metastasis, vascular infiltration, tumor status, and stage. Therefore, the proportion of ctDNA varies greatly, ranging from < 0.1% to > 90% [24].

**Fig. 4:**
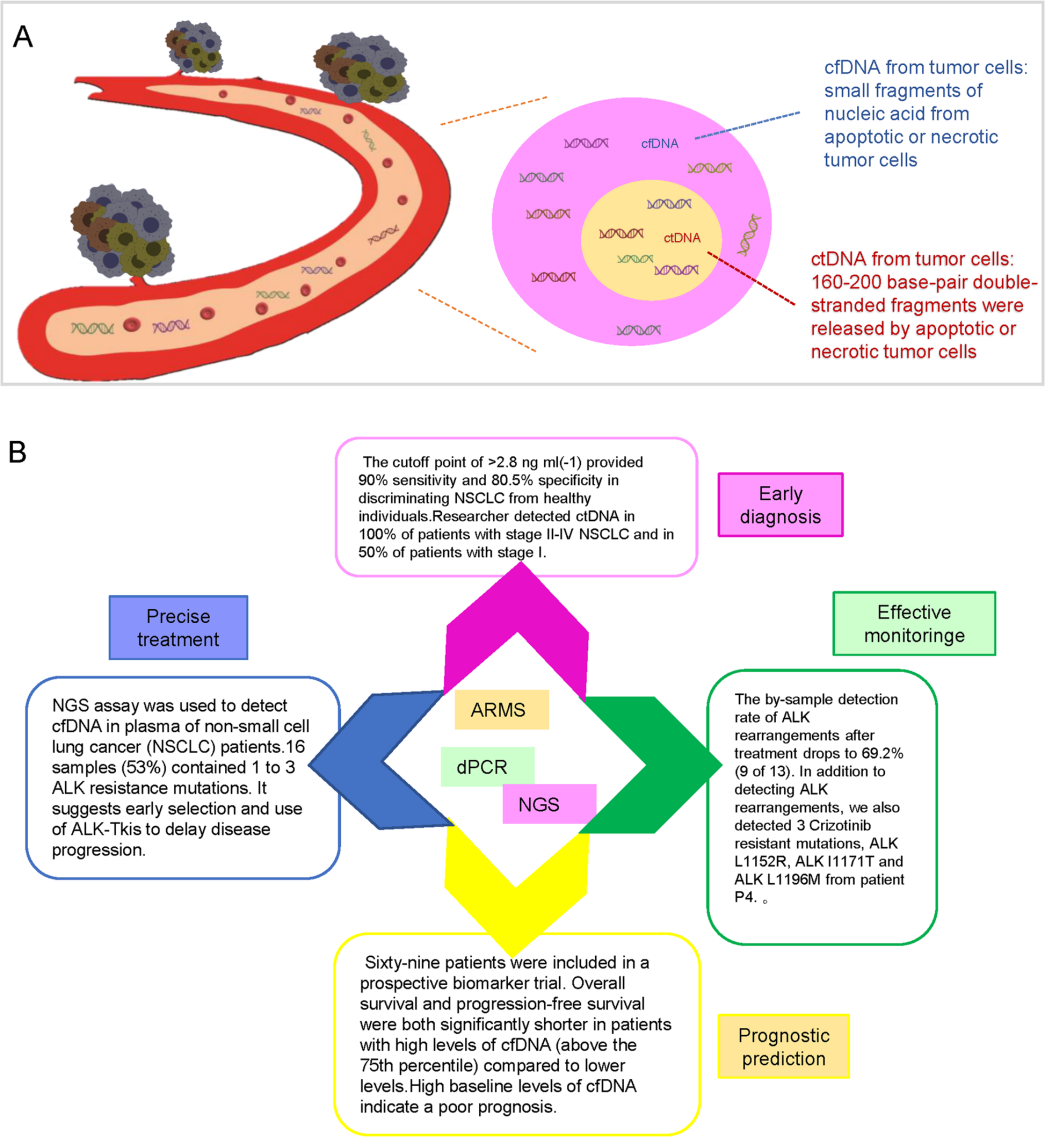
Clinical application and common detection techniques of ctDNA in lung cancer. **A** Circulating free DNA (cfDNA) from both normal and tumor cells is released via necrosis and apoptosis into the blood circulation. In cancer patients, a small portion of cfDNA is shed into the blood by tumor cells—this is called ctDNA. **B** ctDNA is cleared soon after entering the circulation due to its short half-life of 2 h, allowing for non-invasive real-time tumor monitoring. The implementation of ctDNA detection in clinical practice holds great potential for early detection and personalized medicine in lung cancer

Although the proportion of ctDNA tends to be parallel to the tumor load in a single patient, great differences are observed in patients with the same cancer type, which may reflect differences in biological variation or cell mortality in a single tumor. Compared with traditional tumor biopsy, collecting ctDNA from blood is non-invasive and can be repeated over time, which may contribute to the early diagnosis of cancer and the identification of small residual diseases or recurrence rate, in addition to monitoring treatment response in real-time and predicting prognosis [25]. At present, ctDNA detection technologies mainly include amplification refractory mutation system (ARMS), digital PCR, and NGS, all of which can perform qualitative and quantitative analysis of ctDNA and accurate detection of ctDNA through DNA amplification (Fig. 4).

When evaluating the potential clinical value of plasma ctDNA levels as a diagnostic tool for early lung cancer, researchers have found that the plasma ctDNA level of patients with NSCLC was significantly higher than that of subjects with chronic respiratory inflammation and healthy individuals. At the same time, when distinguishing NSCLC patients from healthy individuals, the sensitivity and specificity of ctDNA levels were 90 and 80.5%, respectively [26]. Furthermore, in another study ctDNA was detected in 100% of the plasma specimens taken from patients with stage II–IV NSCLC, 50% of the plasma specimens taken from of the patients with stage I NSCLC, and the 96% specificity of its mutant allele fragment decreased to 0.02%. At the same time, it was found that ctDNA level is highly correlated with tumor volume, which can distinguish residual lesions and treatment-related imaging changes, suggesting that measuring ctDNA levels can evaluate the curative effect earlier than imaging methods [15].

In addition to the traditional detection and monitoring method, NGS of plasma ctDNA is used to detect ALK gene rearrangement and fusion genes. This non-invasive detection method has good sensitivity and excellent specificity. It not only detects mutation-mediated drug resistance of newly diagnosed ALK-positive NSCLC patients but also has significance for NSCLC patients with distant metastasis. Studies have shown that capture-based sequencing technology can detect and quantify ALK rearrangement and other somatic mutations in plasma with high sensitivity, including mutation-mediated drug resistance, paving the way for its application in the clinical identification of driving fusion genes, in addition to monitoring tumor dynamics and drug treatment effects. ctDNA detection is used to guide the selection of appropriate anaplastic lymphoma tyrosine kinase inhibitors (ALK-TKIs), which can provide valuable information for the precise drug treatment of lung cancer patients, especially for patients who are not suitable for biopsy [27], greatly improving the clinical treatment efficiency. ctDNA detection also shows high value in early diagnosis and prognosis prediction in patients, providing a new basis and reference for the precise drug treatment plan of lung cancer patients [28].

A previous study evaluated the feasibility of using ctDNA in blood samples as a substitute for tumor biopsy to determine EGFR mutation status and further linked EGFR mutation in ctDNA to prognosis [29]. Univariate analysis of patients with an EGFR mutation in ctDNA showed that the L858R mutation in tumor tissue or ctDNA was a marker of shorter OS and PFS. The median OS of patients with anL858R mutation in ctDNA was 13.7 months, and the median OS of patients without anL858R mutation in ctDNA was 27.7 months. In the multivariate analysis of 76 patients with an EGFR mutation in ctDNA, only erlotinib treatment was an independent predictor of prolonged PFS. Thus, L858R mutation in ctDNA may be a new alternative prognostic marker.

In addition, ctDNA is a very valuable method to predict the risk of post-operative recurrence of NSCLC. In a prospective study that included 69 patients with advanced NSCLC, dynamic changes in ctDNA and plasma mutant KRAS (pmKRAS) in NSCLC patients during treatment were found, and the prognostic value of evaluating the content of ctDNA was also shown. It was found that the overall survival and progression free survival of patients with high levels of ctDNA were significantly shorter than those with low levels of ctDNA. ctDNA above baseline indicated a poor prognosis [30], and it may be more reliable if combined with the dynamic changes of ctDNA [31].

Therefore, gene changes, such as gene mutation, loss of heterozygosity, microsatellite instability, and gene methylation, can be found in the ctDNA of patients with lung cancer [32–34]. Consequently, ctDNA analysis has shown high value in early diagnosis and prognosis. Precise treatment is very important for lung cancer, especially for NSCLC patients with gene mutations. Plasma ctDNA harvested from NSCLC patients using liquid biopsy technology was used to detect the gene mutations of NSCLC patients and identify new genetic changes for acquired drug resistance. Then, suitable TKI was employed for treatment, which greatly improves the clinical treatment efficiency [35].

However, like CTCs, the content of ctDNA in tumor patients is very low and the half-life is short. Easy to be covered up by normal cfDNA makes a need of highly sensitive detection technology [36]. With the rapid development of DNA sequencing technology, the direct qualitative and quantitative analysis of ctDNA can be performed through existing detection methods, such as ARMS, digital PCR, and NGS after DNA amplification. In addition to the standardization of the detection process, the clinical application value of ctDNA also needs to be further verified by a large number of relevant clinical trials [37].

#### Exosomes

Exosomes are a membranous vesicle structures initially found in reticulocytes in the late 1980s [38]. They belong to extracellular vesicles (EVs), with a diameter of 40–160 nm (average diameter of about 100 nm). They are released by different types of cells and originate from the endosome system. The continuous invagination of the plasma membrane eventually leads to the formation of multivesicular bodies, and under the synergistic action of various mechanisms, these vesicles fuse with the cell plasma membrane and are finally secreted. The multivesicular bodies can interact with other intracellular vesicles and organelles, contributing to the diversity of exosome components [39].

EVs are divided into exosomes, oncosomes, microbubbles, and apoptotic bodies according to size, morphology and origin. Exosomes are widely distributed in saliva, blood, bronchoalveolar lavage (BAL) fluid, sputum, and other body fluids [40]. The cell membrane invaginates, forms endosomes, then forms multivesicular bodies, and finally secretes the bodies to the outside of the cell to become exosomes with a diameter of 30–100 nm. Exosomes carry a variety of proteins, lipids, DNA, RNA, and other important information from the cell. Surface markers mainly include CD63, CD81, CD9, TSG101, and HSP27 [41].

**Fig. 5:**
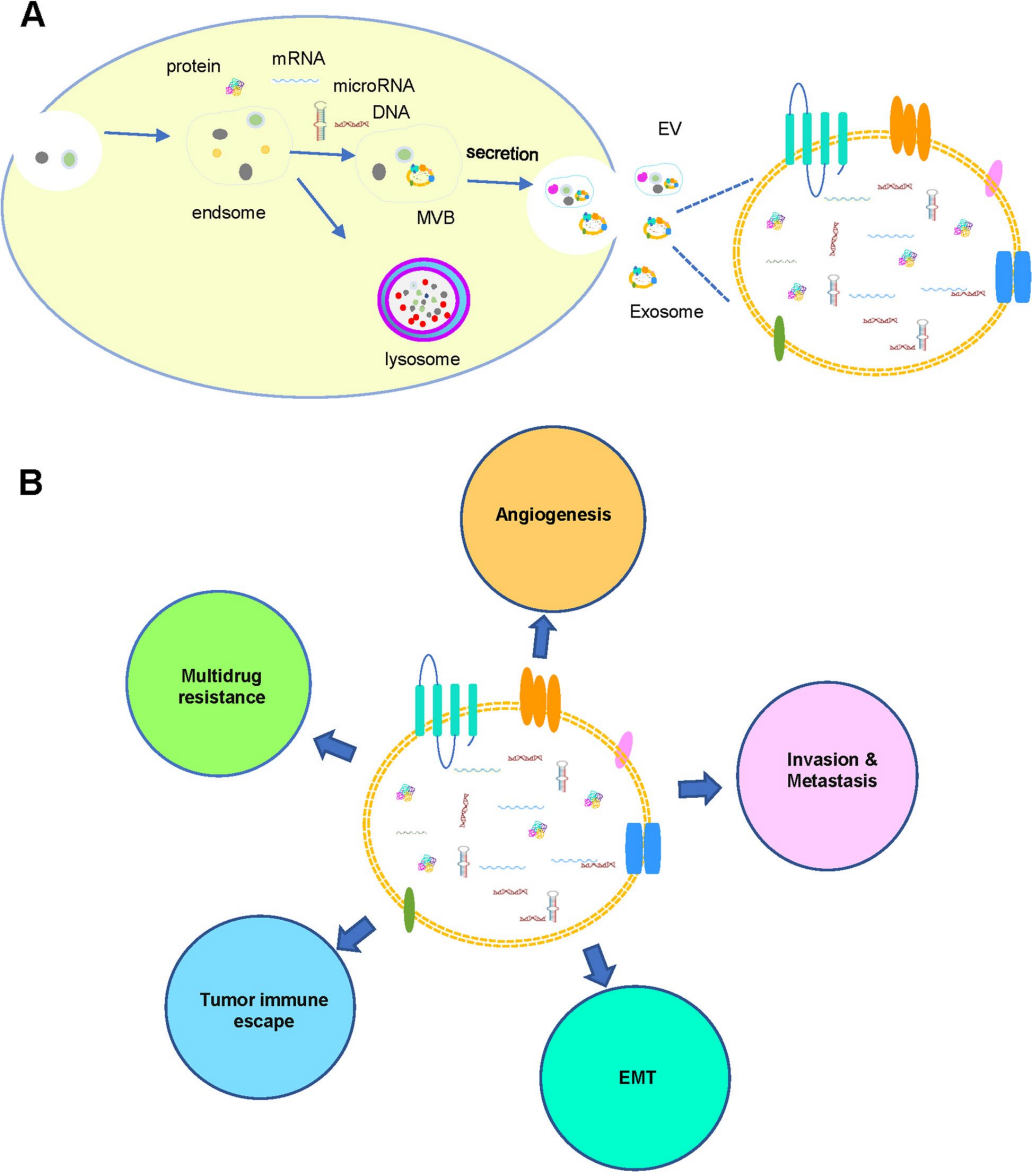
Exosomes as a next-generation diagnostic and therapeutic tool in lung cancer. **A** Exosomes are extracellular vesicles (EVs)released from all cells and play an important role in cellular physiology and pathology, including cancer. **B** With liquid biopsy, exosomes can be isolated and analyzed, providing insights into promising treatment options and serving as a vehicle for nucleic acids or drugs that have anti-neoplastic effects

Exosomes are considered to be possible to remove redundant and/or unnecessary components from cells to maintain intracellular homeostasis. They can also be used as a tool for intercellular material and information exchange and as a carrier for intercellular information transmission to transfer information molecules such as DNA, noncoding RNA (including miRNA and long-chain noncoding RNA (lncRNA)), mRNA, and protein. Exosomes are self-regulating, and can also regulate adjacent cells and distant target cells by autocrine or paracrine mechanisms, and mediate the physiological and/or pathological activities of the body [42]. Exosomes are involved in angiogenesis, EMT, invasion and metastasis, immune escape, drug resistance, and other aspects in the occurrence and development of tumors and have excellent clinical application prospects (Fig. 5).

In recent years, it has been found that exosomal miRNAs can be used as an important medium for information exchange between lung cancer cells and other cells, and they play an important role in the pathological process of tumors [43]. It has been suggested that exosomes can be used for the early diagnosis, prognosis, and drug resistance evaluation of tumors. Understanding exosomes and miRNAs in exosomes may be the key to understanding lung cancer. Inhibiting the formation and release of exosomes may be a potential new strategy for the treatment of lung cancer. Exosomes effectively maintain the stability nucleic acid substances, thereby protecting ctDNA from degradation, which allows for the supplementary analysis of ctDNA and miRNA found in exosomes.

Studies have confirmed that secreted miRNAs (mir-151a-5p, mir-629, mir-200b-5p, mir-100, mir-154-3p, and mir-30a-3p) can distinguish between patients with LUAD and patients with pulmonary granuloma [44]. Secreted microRNAs (mir-379, mir-378a, mir-139-5p, and mir-200b-5p) can be used to distinguish patients with lung cancer from healthy people, which suggests that secreted miRNAs in the BAL fluid can be used to identify patients with lung cancer [18]. At the same time, secreted miRNAs can also be used as biomarkers to identify different types of NSCLC. Further studies have confirmed that plasma exosomes containing mir-30e-3p, mir-30a-3p, mir-181-5p, and mir-361-5p are specific diagnostic biomarkers of adenocarcinoma, while plasma exosomes containing mir-15b-5p, mir-10b-5p, and mir-320b can be used as specific diagnostic biomarkers of squamous cell carcinoma [45].

A previous study investigated the regulatory effect of exosomes on the sensitivity of the lung cancer cell line A549 and found that the secretion of exosomes increased after adding cisplatin (DDP) to A549 cells [46]. Furthermore, adding the secreted exosomes to the media of other A549 cells increased the resistance of these A549 cells to DDP. After A549 cells were exposed to DDP, the expression level of several miRNAs and mRNAs related to DDP sensitivity in the exosomes changed significantly. These changes mediated the resistance of A549 cells to DDP, which suggests that inhibiting the formation and release of exosomes may increase the sensitivity of lung cancer cells to DDP and may provide a new strategy for lung cancer treatment in the future [47]. In another study that investigated the role of secreted miRNAs in regulating cisplatin resistance in NSCLC cells, it was shown that the level of miR-4443 in cisplatin-resistant NSCLC tumor tissue-derived exosomes was upregulated compared with cisplatin-sensitive tissue-derived exosomes. Drug-resistant exosomes produce cisplatin resistance by transferring miR-4443 to sensitive cells [48]. Another study investigated the relationship between the level of secreted miR-146a-5p and the sensitivity of cisplatin chemotherapy in NSCLC and found that the recurrence rate of patients with advanced NSCLC with a low level of serum miR-146a-5p was higher than that of patients with a high level of serum miR-146a-5p [49]. The expression of miR-146a-5p and exosomes in DDP-treated A549 cells was reported to be higher than that in A549 cells without DDP treatment. Additionally, during cisplatin-induced drug resistance, the expression of mir-146a-5p in NSCLC cell lines or exosomes decreased gradually, while overexpression of mir-146a-5p was shown to reverse DDP resistance in A549 cells. These findings suggested that inhibition of exosome formation and release may be a potential new strategy for the treatment of lung cancer [50].

It is generally believed that exosomes are invagination of the cell membrane, the formation of endosomes, and then the formation of multivesicles [51]. The fusion of multivesicles with the plasma membrane leads to the release of intraluminal vesicles [52]. Effective extraction is the key step for experimental research. At present, the extraction and purification methods of exosomes mainly include density-based separation, precipitation, size-based separation, surface component affinity-based separation, electrodynamics separation, and microfluidic chips [53]. As a promising biomarker for cancer diagnosis, exosomes have attracted much attention in cancer liquid biopsies, but their clinical limitations are also affected by the richness of exosomes, separation instruments, and purity [54]. Although domestic and foreign scholars have made some progress in trying different separation and purification methods, cost and efficiency are still limiting the research and application of exosomes. Therefore, the establishment of a reliable exosome extraction method would be conducive to the clinical application of exosomes [55].

#### Other liquid biopsy components

Peripheral blood-circulating RNA refers to extracellular RNA, including mRNA, miRNA, lncRNA, and other types of RNA, actively secreted by cells and/or released after apoptosis into blood or other bodily fluids. miRNA is an endogenous, non-coding RNA with regulatory functions in eukaryotes, with a size of about 18–25 nucleotides [56]. Mature miRNAs form RNA-induced silencing complexes, recognize target mRNAs by base complementary pairing, and guide silencing complexes to inhibit the translation of target mRNAs or degrade target mRNAs according to different degrees of complementarity [57]. Thus, miRNAs regulate the expression of various oncogenes and tumor suppressor genes.

At present, the detection of miRNA markers is of vital significance for the early diagnosis of lung cancer. Let-7a is one of the most studied biomarkers at present. Studies have shown that the expression level of let-7a in the serum of NSCLC patients is significantly lower than that of healthy controls [58]. Using real-time quantitative polymerase chain reaction detection, it was also found that there is a statistically significant difference in the expression of miR-125 in the serum of NSCLC patients and healthy people, with a sensitivity of 82% and a specificity of 77% [59]. Studies have found that there are dynamic changes in the expression of miRNA during the early stage versus the entire progression of lung cancer [60]. Some studies have found that inhibiting miR-21 and miR-200b increases the sensitivity of tumor cells to gemcitabine increases, while inhibiting miR-141 is inhibited reduces the growth rate of tumor cells [61, 62]. Therefore, it is speculated that miRNA is involved in the regulation of tumor chemotherapeutic drug sensitivity. miRNA has been considered to be used as a biological index for the early detection, risk assessment, and early diagnosis of lung cancer [63–65].

With sequencing technology development, a huge number of lncRNAs have been identified. LncRNAs, originally considered as junk RNA, are reported to dysregulate in various types of cancer [66]. Hu et al. [67] found that lncRNA H19 was significantly increased in the plasma of patients with lung cancer, making it a potential biomarker for the auxiliary diagnosis of lung cancer. lncRNA MIR22HG has been shown to function as a tumor suppressor and has potential as a new therapeutic target for lung cancer [68]. lncRNA p53-induced transcript (LINC-PINT), an lncRNA that functions as a tumor suppressor gene, is found to be involved in various tumors and malignant activities [69]. LINC-PINT is also found to be downregulated in lung cancer. Furthermore, decreased expression of LINC-PINT predicts poor prognosis and advanced clinical tumor stages, suggesting that lncRNAs could serve as a diagnostic and prognostic indicator for lung cancer patients.

Unlike linear RNA, circular RNA (circRNA) has no 5′ end cap structure and 3′ end poly (A) tail and exists in the structure of a covalently closed loop [70]. circRNA has a variety of functions, including functioning as a miRNA sponge, regulating transcription between parental genomes, intervening splicing, and interacting with RNA-binding proteins. Some circRNAs participate in gene expression regulation through the proteins they encode. As a new RNA molecule, circRNA can be continuously and stably expressed in cells because of its unique closed-ring structure and high stability [71]. It is likely to become a biomarker for the early diagnosis and prognosis of lung cancer. circRNA is involved in the occurrence and development of tumors and plays a role in promoting or inhibiting tumor growth. A previous study has verified that circFASRA in plasma has diagnostic value for NSCLC [72]. By detecting circFASRA in the plasma of NSCLC patients and normal people, it can be found that circFASRA in the plasma has diagnostic value and can be used as a biomarker for the detection of non-invasive NSCLC [73]. With the development of second-generation sequencing technology, screening circRNA differentially expressed in body fluid samples of lung cancer patients and normal controls can be used not only as a candidate marker for diagnosis/prognosis but also as a potential therapeutic target [74].

TEPs are tumor cells that transfer tumor-related molecules to platelets and change their RNA and protein to become TEPS [75]. TEPS are involved in the progression and diffusion of a variety of solid tumors. Spliced TEP RNA proxy markers can provide specific information about the existence, location, and molecular characteristics of cancer and promote tumor cell growth and dissemination, but the specific mechanism is not clear [76]. A previous study used a particle swarm optimization enhancement algorithm to effectively select RNA biomarkers from a platelet RNA sequencing library [77]. The accuracy of early and advanced NSCLC based on TEPs was 81 and 88%, respectively. Another study analyzed the TEP RNA from the blood of nine patients with NSCLC (stage I and II) and eight healthy controls by RNA sequencing as a way to detect phase I NSCLC. It was found that the level of platelet ITGA2B in patients with NSCLC was significantly higher than that in the control groups. ITGA2B combined with carcinoembryonic antigen and stage can be used as survival predictors. It has been suggested that TEP ITGA2B is a promising marker, which can improve the identification of patients with stage I NSCLC and distinguish between malignant and benign pulmonary nodules [78].

Vascular endothelial cells in peripheral blood are called circulating endothelial cells (CECs),which include mature endothelial cells derived from existing vascular walls and endothelial progenitor cells or endothelial progenitor cells (EPCs) derived from bone marrow [79]. The formation modes of tumor neovascularization mainly include angiogenesis and vasculogenesis. The former refers to the “sprouting” of existing blood vessels as endothelial cells, while the latter refers to bone marrow EPCs entering the peripheral blood, proliferating, and differentiating into mature endothelial cells and forming primitive vascular plexus. These two forms depend on CECs—endothelial cells that fall off the original vascular wall or EPCs produced by bone marrow mobilization, which differentiate into CECs in the peripheral circulation, proliferate, adhere, and form a tubular structure to form a new blood vessel for the tumor [80]. Angiogenesis is a prerequisite for the proliferation and differentiation of malignant tumors. Many tumor-derived endothelial cells (TECs) enter the blood and transform into CTECs [81].

Studies have shown that CTECs may become a method for early diagnosis and chemotherapy efficacy in patients with early NSCLC [82]. Using subtraction enrichment-immunofluorescence in situ hybridization (SE-iFISH) quantitative analysis, it was found that counting the total number of CTCs and CTECs may help to identify malignant nodules with high sensitivity, while quantifying tetraploid CTCs and CTECs may help to identify malignant nodules with high specificity [83]. The results showed that the combined detection of specific subtypes of aneuploid CD31^+^ CTECs and CD31^−^ CTCs is helpful in effectively identifying malignant nodules in patients with early NSCLC [84]. The level of CECs in partial response (PR) patients was found to be significantly higher than that of stable disease (SD) or progression disease (PD) patients. In terms of treatment effect, the CEC count in PR patients after chemotherapy was significantly lower than that at baseline. In contrast, the CEC level of PD patients after chemotherapy was significantly higher than that at diagnosis. There was no significant change in the amount of CECs in SD patients before and after treatment. This suggests that the CEC level may be an early predictor of treatment effects in patients with advanced NSCLC [85]. The application of CTECs in the early diagnosis, efficacy detection, and prognosis prediction of lung cancer patients is still in the research stage, and more clinical validation is needed. It is believed that further research to determine and optimize the specific markers of CECs is of great significance to determine the early diagnosis and chemotherapy efficacy of lung cancer patients.

## Conclusions

Liquid biopsy opens up a new way for the early screening, diagnosis, and treatment of lung cancer, especially when tissue samples cannot be obtained. Using circulating biomarkers and liquid biopsy in lung cancer allows assessing the instantaneous molecular, genetic and epigenetic profile of cancer cells selected as drug-resistant clones from previous therapies. Circulating biomarkers could be non-invasive tools able to evaluate the response to ongoing treatments, thus rapidly guiding the medical choice for eventual further chemotherapy cycles or the need to change treatment strategy. However, it is necessary to further develop biomarkers that are highly sensitive and specific for the early diagnosis of lung cancer. Besides, an exhaustive prognostic biomarker has not yet been identified. Furthermore, the lack of standardized detection methods, the scarce accessibility in the territory, the high costs, and the uncertainty about cut-off levels could hinder the use of circulating biomarkers in routine clinical practice. More sufficient evidence is needed to support the transformation of liquid biopsy from the research stage to clinical use, but its potential in the diagnosis and treatment of lung cancer cannot be ignored. Further advanced molecular biological detection methods to enhance the reliability and practicality of liquid biopsy are needed before it can be widely used in clinical practice. In conclusion, in the future, liquid biopsy technology is expected to play a greater role in the early diagnosis, accurate drug use, dynamic monitoring, and prognosis evaluation of lung cancer.

## Data Availability

The datasets supporting the conclusions of this article are included within the article.

## Abbreviations

ALK: Anaplastic lymphoma kinase
ALK-TKI: Anaplastic lymphoma tyrosine kinase inhibitor
ARMS: Amplification refractory mutation system
BAL: Bronchoalveolar lavage
cfDNA: Cell free DNA
circRNA: Circular RNA
ctDNA: Circulating tumor DNA
CTC: Circulating tumor cells
CTEC: Tumor vascular endothelial cell
EMT: Epithelial mesenchymal transformation
EPCs: Endothelial progenitor cells
EVs: Extracellular vesicles
FISH: Fluorescence in situ hybridization
HPD: Hyper progressive disease
ICIs: Immune checkpoint inhibitors
LDCT: Low-dose spiral CT
lncRNA: Long-chain noncoding RNA
LUAD: Lung adenocarcinoma
LUSC: Lung squamous cell carcinoma
miRNA: microRNA
NGS: Next generation sequencing
NSCLC: Non-small cell lung cancer
pmKRAS: Plasma mutant KRAS
SCLC: Small cell lung cancer
TEP: Tumor platelets
